# Construction and validity of educational technology about the human papillomavirus vaccine for adolescents

**DOI:** 10.1590/0034-7167-2023-0048

**Published:** 2023-12-08

**Authors:** Paula Gisely Costa Silva, Ilma Pastana Ferreira, Lidiane Assunção de Vasconcelos, Haroldo Gonçalves de Jesus, Tobias Ferreira Gonçalves, Ivonete Vieira Pereira Peixoto

**Affiliations:** I Universidade do Estado do Pará. Belém, Pará, Brazil

**Keywords:** Human Papillomavirus Viruses, Immunization, Adolescent, Educational Technology, Graphic Novel, Virus del Papiloma Humano, Inmunización, Adolescente, Tecnología Educacional, Novela Gráfica, Papilomavírus Humano, Imunização, Adolescente, Tecnologia Educacional, História em Quadrinhos

## Abstract

**Objectives:**

to construct and validate an educational technology in comic book format about the human papillomavirus vaccine aimed at adolescents.

**Methods:**

a methodological study, with a quantitative approach, through the agreement method. It was carried out in two phases: educational technology construction and content validity. Participants are expert judges in the health field. Data collection took place in a virtual environment, through a questionnaire. Data analysis was performed by calculating the Content Validity Index. A Content Validity Index of at least 80% was accepted.

**Results:**

the comic book’s overall Content Validity Index was 82%, reaching the minimum limit established to be validated.

**Conclusions:**

comics are fundamental in the teaching-learning process, aiming to catch adolescents’ attention. Therefore, it is characterized as a valid tool to inform, in a playful manner, about the human papillomavirus vaccine.

## RESUMO

**Objetivos:**

construir e validar uma tecnologia educacional em formato de história em quadrinhos sobre a vacina contra o papilomavírus humano voltada para adolescentes.

**Métodos:**

estudo metodológico, com abordagem quantitativa, através do método de concordância. Realizado em duas fases: construção de tecnologia educacional e validação de conteúdo. Os participantes são juízes especialistas da área da saúde. A coleta de dados ocorreu em ambiente virtual, através de questionário. A análise de dados foi realizada através do cálculo do Índice de Validade de Conteúdo. Foi aceito um Índice de Validade de Conteúdo de no mínimo 80%.

**Resultados:**

o Índice de Validade de Conteúdo global da história em quadrinhos foi de 82%, alcançando limite mínimo estabelecido para ser validado.

**Conclusões:**

a história em quadrinhos é fundamental no processo ensino-aprendizagem, visando cativar a atenção de adolescentes. Portanto, caracteriza-se como uma ferramenta válida para informar, de maneira lúdica, sobre a vacina contra o papilomavírus humano.

## RESUMEN

**Objetivos:**

construir y validar una tecnología educativa en formato cómic sobre la vacuna contra el virus del papiloma humano dirigida a adolescentes.

**Métodos:**

estudio metodológico, con enfoque cuantitativo, mediante el método de concordancia. Se realizó en dos fases: construcción de tecnología educativa y validación de contenidos. Los participantes son jueces expertos en el campo de la salud. La recolección de datos se realizó en un ambiente virtual, a través de un cuestionario. El análisis de los datos se realizó mediante el cálculo del Índice de Validez de Contenido. Se aceptó un Índice de Validez de Contenido de al menos el 80%.

**Resultados:**

el Índice de Validez de Contenido general del cómic fue del 82%, alcanzando el límite mínimo establecido para ser validado.

**Conclusiones:**

las historietas son fundamentales en el proceso de enseñanza-aprendizaje, teniendo como objetivo cautivar la atención de los adolescentes. Por ello, se caracteriza como una herramienta válida para informar, de forma lúdica, sobre la vacuna contra el virus del papiloma humano.

## INTRODUCTION

The human papillomavirus (HPV) is a virus that affects the epithelium, inducing the formation of lesions on the skin and mucous membranes, especially in the anogenital region. It is related to several types of cancer, such as penile, prostate, anus, oropharynx, cervix and vulva cancer. HPV is transmitted as a result of unprotected sexual activity, and may rarely occur during childbirth and through the use of fomites^(1)^.

There are more than 200 HPV genotypes; of these, approximately 40 affect the anogenital tract^(2)^ and can be divided into two groups: low oncogenic risk or high oncogenic risk. High oncogenic risk serotypes are related to the appearance of several types of cancer, such as cervical cancer, where types 16 and 18 are responsible for almost 70% of cases. Meanwhile, low-risk oncogenic serotypes, types 6 and 11, are linked to about 90% of genital warts^(1)^.

Thus, it is essential to prevent this condition through the use of condoms and vaccination, in addition to early diagnosis by performing a cytopathological examination of the cervix, indicated for women aged 25 to 64 years who have already started sexual activity.

Currently, the quadrivalent vaccine (types 06, 11, 16 and 18) is available for girls and boys aged 9 to 14 years^(3)^, with two doses with an interval of six months. The vaccine also covers people living with the human immunodeficiency virus (HIV), transplanted solid organs, bone marrow or cancer patients of both sexes, between 9 and 45 years old, with 3 doses with an interval of 0, 2 and 6 months^(4)^.

In view of this, it is essential to use different strategies that promote vaccination coverage compliance and expansion, such as the use of educational technologies, such as booklets, videos and comics, which aim to collaborate in teaching-learning^(5)^.

In this perspective, comic books are educational technologies that present texts accompanied by sequential illustrations, shown in a clear and objective manner, favoring readers’ imagination. Thus, they become a fundamental element to catch children’s and adolescents’ attention, making them absorb the knowledge approached^(6)^.

After a broad search in the literature, it was found that, among the educational technologies already produced about HPV and HPV vaccine, the comic book format was cited only once, evidencing the predominance of educational video format.

Therefore, the need for this study is justified by the social relevance presented, considering the association between knowledge about factors related to HPV and compliance with the vaccine. The final version of the comic book will subsidize educational actions aimed at adolescents in schools and health services about HPV vaccine.

## OBJECTIVES

To construct and validate an educational technology in the form of comic books about HPV vaccine aimed at adolescents.

## METHODS

### Ethical aspects

The project was approved by the Research Ethics Committee of the *Universidade do Estado do Pará*, following Resolutions 466/12, 510/16 and 580/18 of the Brazilian National Health Council. All expert judges consented to participate in the study via the digital platform. The judges were informed that they could withdraw from the research at any time. The participating judges’ anonymity and confidentiality were guaranteed.

### Study design, sample and period

This is a methodological study, with a quantitative approach, using the agreement method. It was carried out in two phases: comic book educational technology construction based on a literature review (phase 1); and content validity (phase 2).

Educational technology construction (phase 1) was carried out through an integrative literature review (ILR). The question formulated to guide IRL, through the PICo strategy, was: what is the summary of knowledge of adolescents about HPV and HPV vaccine in Brazil? “P” corresponds to the population (adolescents); “I” corresponds to the phenomenon of interest (knowledge about HPV and HPV vaccine); and “Co” corresponds to the context of the study (Brazil). The search for articles was carried out in the Virtual Health Library (VHL) in three databases, as Latin American and Caribbean Literature in Health Sciences (LILACS), Medical Literature Analysis and Retrieval System (MEDLINE) and Nursing Database (BDENF), through Health Sciences Descriptors (DeCS), using the following search strategy: (“*adolescente*” OR “adolescent”) AND (“*conhecimento*” OR “knowledge”) AND (“*imunização*” OR “*Vacinas*” OR “immunization”) AND (“*papillomavirus humano*” OR “human papillomavirus”), from May to June 2022.

To methodologically subsidize the comic book construction, the adapted steps of the process of educational health technology elaboration, described by Echer^(7)^ and Santos Júnior^(8)^: Step 1. Creative step and Step 2. Data architecture.

In the creative step (step 1), substep 1.1, content elaboration, it was decided to address what is HPV; how it is transmitted; prevention methods; what is HPV vaccine; vaccination schedule; vaccine age group; adverse effects; relationship between HPV and cancer. In substep 1.2, character and scenario definition, a nurse, five adolescents and a mother were established as characters. The chosen scenarios were a classroom and a Basic Health Unit (BHU).

In substep 1.3, plot construction, the comic book addressed adolescents debating about HPV; modes of transmission and prevention; about HPV vaccine, covering vaccination schedule and interval between doses; vaccine age group; and the relationship between HPV and cancer. Posteriorly, adolescents were at the BHU to receive the HPV vaccine and talked to the nurse about the vaccine. Finally, they appear talking to each other about the importance of taking the vaccine.

Step 2, data architecture, with its respective substeps, such as 2.1 layout, 2.2 design, 2.3 assembly, 2.4 colorization, 2.5 final art, 2.6 finishing and 2.7 review, were constructed with the help of a graphic designer.

Content validity (phase 2) was performed in a virtual environment via Google Forms. The research sample consisted of a group called expert judges in the health area, responsible for judging content, such as objectives, structure and presentation, and relevance. The survey of judges and data collection were carried out from September to November 2022.

**Figure 1:**
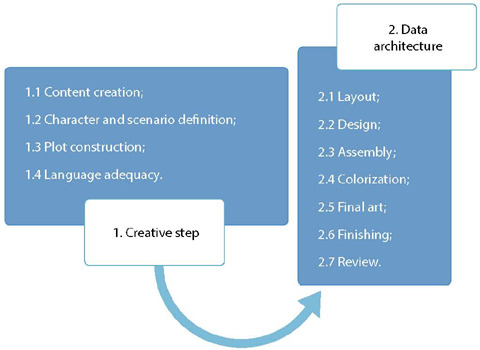
Steps of educational technology construction in comic book format

For the selection of expert judges, six inclusion criteria were established, based on Benevides^(9)^: care experience with the subject of the study; have scientific production on construction and validity on the subject; scientific production on the topic addressed; having a doctoral degree, a master’s degree or specialization in the area covered. Thus, a search for curricula was carried out on the *Plataforma Lattes*, using the “search by subject” tool, with the keywords “immunization” and “educational technology”. They were also selected using the snowball method. According to Costa^(10)^, the snowball method or non-probabilistic approach is when:

Initially, the researcher specifies the characteristics that the sample members should have, then identifies a person or a group of people congruent with the necessary data, then presents the study proposal and, after obtaining/recording such data, requests that the participant(s) of the research nominate(s) other person(s) belonging to the same target population.

Judges were invited to participate in the research, through an invitation letter sent through the “contact” section of the *Plataforma Lattes*. The judges who accepted were sent a Google Forms link containing the Informed Consent Form, educational technology and assessment instrument. Participants were given a maximum period of 15 days to return the material, counting from material delivery.

A total of 45 judges were invited. Among these, 12 agreed to participate in the research. However, after sending the material, only 9 expert judges responded to the instrument, corroborating Pasquali^(11)^, who says it is necessary to have from 06 to 20 judges to validate a technology. It should be noted that the research is within the number of judges nominated.

### Result collection and analysis

The instrument used in the collection of data for content validity by expert judges in the health area was a questionnaire prepared by the author, based on Teixeira’s instrument^(12)^. It is divided into 02 parts: part I contains questions related to the profile of expert judges; part II is related to objectives, structure and presentation, and, finally, relevance.

For comic book validity by expert judges in the health area, the Content Validity Index (CVI) was calculated, which demonstrates agreement on the assessed aspects. A CVI of at least 80% (0.80) was accepted. For this calculation, a Likert-type scale was used with scores from one to four. The index was calculated through the sum of agreement of items marked as “1” and “2” by experts, divided by the total number of responses.

CVI = Number of responses 1 and 2/Total number of responses

## RESULTS

The results presented in Table 1 correspond to the profile of 9 expert judges in the health area according to gender, age, state, education, time since graduation and title.

**Table 1:** Characterization of expert judges in the health area, Belém, Pará, Brazil, 2022

Variables	n	%
Gender		
Female	9	100%
Age		
20 to 39 years	4	44.5%
40 to 59 years	3	33.3%
≥ 60 years	2	22.2%
State		
Brazilian states	8	88.9%
States outside Brazil	1	11.1%
Training		
Nursing	9	100%
Time since graduation		
1 to 10 years	3	33.3%
11 to 20 years	4	44.5%
>20 years	2	22.2%
Degree		
Master’s	3	33.3%
Doctoral	3	33.3%
Postdoc	3	33.4%

As for the profile of expert judges in the health area, 100% are female; age remained between 27 and 64 years; of these, 44.5% are aged between 20 and 39 years. All regions of Brazil were represented in the study, highlighting the North region, where all judges are from the state of Pará, and a state outside Brazil, located in Australia. Regarding training, it is noteworthy that 100% are in the nursing category. Time since graduation remained between 3 and 42 years, with emphasis on the variable between 11 and 20 years (44.5%). Degrees ranged from master’s (33.3%), doctoral (33.3%) and postdoc (33.4%).

The results presented in Table 2 correspond to the frequency of scores obtained in each item per chunk and the respective CVI result. It is worth noting that some items were answered by only 8 judges, given judges’ freedom not to answer questions that cause them embarrassment or that they do not know how to answer.

**Table 2:** Frequency of scores obtained in the assessment of expert judges in health regarding validity criteria, Belém, Pará, Brazil, 2022

Item	Validity				
	1	2	3	4	CVI
Objectives					
1.1 Content is consistent with the target audience’ needs	6	2	1	0	88.9%
1.2 Content is important for the target audience’s quality of life	7	2	0	0	100.0%
1.3 Content encourages behavior changes	5	3	1	0	88.9%
1.4 Content can circulate in scientific circles of the area	6	0	2	0	75.0%
1.5 Content is satisfactory for promoting knowledge about HPV and the vaccine	7	1	1	0	88.9%
1.6 Content meets the objectives of institutions working with the target audience	6	2	1	0	88.9%
Structure and presentation					
2.1 Comic book is appropriate for the target audience	6	3	0	0	100%
2.2 Speeches are presented in a clear and objective manner	4	2	3	0	66.7%
2.3 Information presented is scientifically correct	7	1	1	0	88.9%
2.4 Comic book is appropriate to adolescents’ sociocultural level	4	3	2	0	77.8%
2.5 There is a logical sequence of exposed content	6	2	1	0	88.9%
2.6 Information is well structured in concordance and spelling	5	1	3	0	66.7%
2.7 Writing is attractive	4	3	2	0	77.8%
2.8 Cover and title page information are consistent	5	1	3	0	66.7%
2.9 Size of title and topics is adequate	5	1	2	0	75.0%
2.10 Illustrations are expressive and sufficient	4	0	5	0	44.4%
2.11 Page number is adequate	6	2	1	0	88.9%
Relevance					
3.1 Comic book themes portray key aspects that must be reinforced	7	2	0	0	100.0%
3.2 Comic book proposes knowledge construction for adolescents	7	1	1	0	88.9%
3.3 Comic book is adequate for use by other health professionals	7	0	2	0	77.8%

*1 - totally adequate; 2 - adequate; 3 partially adequate; 4 - inadequate; CVI - Content Validity Index; HPV – human papillomavirus.*

The first chunk, with six items, obtained a total of 53 responses. Thus, 88.7% were classified as (1) fully adequate or (2) adequate and 11.3% as (3) partially adequate or (4) inadequate. It was found that, of the six items in the first chunk (objectives), five were considered valid (1.1, 1.2, 1.3, 1.5 and 1.6), reaching a CVI equal to or greater than 80% (0.80). Of the items in the first chunk, it was found that only item 1.4 (content can circulate in the scientific environment of the area) resulted in a CVI of 75%, which is lower than the established limit. Judges considered the comic book with regionalized speeches, which could hinder circulation in other regions of Brazil. Another point would be the need to explain what warts are and make a presentation of the comic book.

The second chunk, referring to structure and presentation, contained eleven items that totaled 98 responses. Among these responses, 76.5% were considered (1) fully adequate or (2) adequate and 23.5% as (3) partially adequate or (4) inadequate. Among the eleven items, seven did not reach the established CVI, and four did (2.1, 2.3, 2.5 and 2.11). The items in this chunk were the ones that showed the greatest disagreement among judges, involving aspects such as speeches, comic book appropriate to the sociocultural level of adolescents, concordance and spelling, attractive writing, consistent cover and title page information, adequate title size and font, and expressive illustrations.

Therefore, these aspects were the ones that suffered the most changes. Judges suggested bringing technologies to the cover to catch readers’ attention; in this chunk, it was also suggested to reduce slang and regional words. Another suggestion was to bring more phenotypic variations, for better social inclusion.

The last chunk, referring to relevance, contained three assessment items, and obtained 27 responses. Thus, 88.9% were assessed as (1) fully adequate or (2) adequate and 11.1% (3) as (3) partially adequate or (4) inadequate. Only item 3.3 (comic book is adequate for use by other health professionals) was not validated, as it reached a CVI of 77.8%, reinforcing issues of speech and regional slang.

The comic book’s overall CVI was 82%, reaching the minimum threshold established for validation. However, there was a need to re-elaborate the material both in terms of lines and illustrations, design and cover, to encourage adolescents to protect themselves against HPV. Given the above, the final version of the comic book consisted of 10 pages. The comic book was composed of redrawn illustrations based on judges’ recommendations.

**Figure 2:**
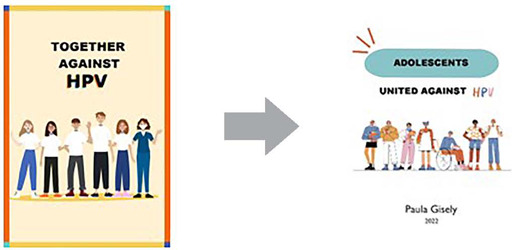
First and second version of the comic book cover

## DISCUSSION

Educational technology construction was carried out in the format of a comic book about HPV vaccine for adolescents, focusing on the strategy of using visual narratives as a facilitating tool for health education processes for different audiences.

Within this perspective, this paper discusses comic content validity by expert judges. In the assessment of study participants, the material produced was considered validated in terms of content, obtaining an overall CVI of 82%. It should be noted that the expert judges who validated the content are, in their entirety, nurses; This data corroborates other studies on educational material validity, justified by the fact that, in general, nurses are the professionals who play the role of educator and have a relevant role in educational technology and intervention application^(13, 14)^.

In participant selection, all regions of Brazil were covered, allowing a comprehensive look at the comic book, with a view to disseminating knowledge about vaccination as a method of preventing HPV. Thus, admitting local-regional differences is essential for building knowledge and educational strategies in accordance with the established reality, since strategic information dissemination in a language accessible to all favors better compliance with vaccination by adolescents, as being vaccinated is crucial for promoting individual and collective health and well-being^(8, 9, 10, 11, 12, 13, 14, 15)^.

According to the results obtained in the first chunk, it is understood that the development of educational materials must be worked on according to the target audience’s needs in a clear and objective manner and that encourages changes in adolescents’ behavior. In this regard, the popularization of the theme addressed in the material also acquires a social commitment, when considering the points raised in the comic book’s plot that culminate in the visual representation of adolescents receiving HPV vaccine, emphasizing the importance of immunization. Thus, it is understood that the material reaches the potential of triggering a critical awareness of oneself, others and the world^(16)^.

It is known that lack of knowledge is one of the factors that influence non-compliance with vaccination by adolescents^(17)^. Therefore, it is necessary to use educational technologies that offer guidance with expressive illustrations, clear, understandable and attractive language for all social levels^(15)^. This statement reinforces judges’ comments regarding the second assessment chunk, which obtained items that did not reach the established CVI, by highlighting that the comic book had formal, long and regional speeches, making access to the target audience difficult.

Thus, in accordance with suggested changes, the comic book underwent a process of re-elaboration, editing, review and layout. The topics outlined by experts were related to speech adequacy, and drawing quality and attractiveness. We highlighted the changes in illustrations of educational technology in order to provide representative, accessible, stimulating and convincing material, considering that, from an educational point of view, the language used in scientific dissemination, especially in comics, must be clear and objective, simplifying the contents for the most diverse audiences^(18)^.

Regarding educational technology validity, judges considered the tool relevant, as it portrays themes that should be reinforced and helps in building knowledge about HPV vaccine among adolescents, confirming what the authors say about health professionals prioritizing health education aimed at the population’s needs, using strategies that favor the population’s interest and understanding on the subject^(19)^.

### Study limitations

As a methodological limitation of this study, it is pointed out that data collection was carried out virtually, since there was no absolute control by the researcher in meeting the established deadlines. However, this strategy allowed for the participation of professionals from various regions of the country, in addition to a specialist in the area of health who resides in Australia, providing cultural diversity to the comic book.

### Contributions to nursing and public health

With this study, we hope to have contributed to the development of new technological resources and educational practices, and to the continuity of scientific research in this area, with the adoption of adequate and experimental methodologies that allow health professionals to systematically define and assess the strategies carried out in the area of health education, more specifically in HPV vaccine.

## CONCLUSIONS

It is considered that the comic book, in general, was considered valid by expert judges in the health area, since it obtained an overall CVI of 82%. However, in the assessment per item, it was found that some items did not reach an agreement rate of 80%, causing several changes in language, appearance, speech and layout.

However, it is believed that the comic book’s impact on adolescents could not be measured in this research, since this study did not include the participation of the target audience in the validity process, requiring other studies.

Considering the aspects highlighted by expert judges, text and illustrations were reworked. Suggestions were about replacing expressions and phrases, adding information, language and proofreading, factors considered essential in educational technology production.

Thus, in terms of comic book structure and presentation, judges’ assessment was fundamental, as they highlighted specific issues of balloon format, lines, fonts, sizes and colors. Therefore, some illustrations were redone, adding clarity, expressiveness, interaction and contextualization.

It is concluded that the educational technology, after adaptations, has become a valid tool to be used for adolescents, with the objective of informing, in a playful manner, what HPV is; how it is transmitted; prevention methods; what is HPV vaccine; vaccination schedule; vaccine age group; adverse effects; relationship between HPV and cancer.
